# Characterization of interaction phenomena of electromagnetic waves with metamaterials via microwave near-field visualization technique

**DOI:** 10.1038/s41598-023-45665-4

**Published:** 2023-10-27

**Authors:** Zhirayr Baghdasaryan, Arsen Babajanyan, Barry Friedman, Kiejin Lee

**Affiliations:** 1 Department of Physics, Sogang University, Seoul, 121-742 Korea; 2 Institute of Physics, Yerevan State University, 0025 Yerevan, Armenia; 3 Department of Physics, Sam Houston State University, Huntsville, TX 77341 USA

**Keywords:** Sensors and biosensors, Metamaterials, Characterization and analytical techniques, Microscopy, Imaging techniques

## Abstract

A new practical imaging technique was presented for metamaterial characterization and investigation by visualizations of the magnetic microwave near-field (H-MWNF) distributions on a metamaterial's surface using the method of thermo-elastic optical indicator microscopy (TEOIM). ITO-based transparent and ceramic-based opaque metamaterial structures were designed for magnetic near-field visualization. Depending on the incident microwave field polarization, the TEOIM system allows the characterization of the metamaterial properties and microwave interaction behavior. The working principle of the periodic structures was investigated through numerical simulations, and the obtained results exhibited strong agreement when compared with experimental observations. Moreover, the visualization of the H-MWNF revealed the potential to characterize and evaluate the absorption and transmission properties effectively.

## Introduction

Metamaterials and metasurfaces are essential components of recent electromagnetic research activities. They are being developed intensively for their widespread use in microwave technologies^1–5^. Metamaterials are innately inhomogeneous artificial two or three-dimensional structures that consist of periodically repeated unit cells, and their unique properties are achieved from geometrical shapes rather than material properties. Electromagnetic metamaterials have been developing rapidly, covering the broad electromagnetic spectrum, from microwave to optical wavelengths, enabling a wide range of applications such as invisibility cloaking^6^, subwavelength imaging^7^, perfect absorbing^8–10^, sensing^11,12^, focusing^13,14^, and efficient energy harvesting^15^. Modern simulation techniques are important for accurately designing, analyzing, and investigating complex metamaterial structures. Simulations help uncover the fundamental working principles of microwave and metamaterial geometry interactions. In addition, understanding the complex behavior of these metamaterials requires accurate visualization techniques that can depict the intricate distribution of the microwave near-field. However, the lack of a practical measurement pathway makes it challenging to visually characterize the designed and fabricated metamaterial's properties.

Microwave imaging has significantly progressed, improving resolution, sensitivity, speed, and imaging capabilities. Initially, microwave imaging techniques were limited in spatial resolution and practical applications. Various methods have emerged through technological advancements which enhance microwave imaging capabilities. These include scanning-probe microscopes^16–19^, metamaterial absorbers^20^, and metasurface-based sensors^21^. Scanning-probe microscopes involve moving a probe along the sample surface and detecting the interaction between the sample and the radio-frequency (RF) field^22^. This method allows for high spatial-resolution imaging at the nanoscale^23^. However, it suffers from slow measurement throughput and complex experimental equipment, limiting its practical use. The utilization of metamaterials has furthered the enhancement of microwave imaging systems, resulting in high sensitivity and efficiency in the imaging process^7,24,25^. This approach offers several advantages, including good sensitivity for detection and the ability to operate across a wide range of frequencies. However, challenges are associated with the design complexity of metamaterials, sensitivity to environmental conditions, and spatial resolution limited by unit cell size. Microwave imaging finds applications in various fields. In medicine, it enables the non-invasive detection and characterization of tumors^26,27^. In non-destructive testing, it aids in evaluating material properties^28–30^. It also has uses in security screening for concealed object detection and remote sensing applications, such as environmental monitoring^31^.

Among modern imaging methods, thermo-elastic optical indicator microscopy (TEOIM) is a promising and high-resolution visualization technique that uses the polarized light microscopy method to visualize electromagnetic near-field distribution in a wide range of operating frequencies^32^. The TEOIM stands out as an exceptional imaging technique due to several advantages. These include a wide field of view, an easily configurable setup, the utilization of inexpensive equipment, and an impressive fast measurement throughput, allowing for efficient and rapid data acquisition and analysis. This paper demonstrates the potential of the TEOIM imaging system for metamaterial characterization under microwave radiation by visualizing the magnetic microwave near-field (H-MWNF) distribution. Ceramic-based opaque metamaterial absorbers and indium tin oxide (ITO)—based transparent frequency selective surfaces (FSS) were designed to demonstrate the TEOIM system's prospects in metamaterial applications. Visualizing the H-MWNF distributions using TEOIM makes it possible to gain valuable insights into how the interaction between the metamaterial and the incident microwave field influences their properties. The TEOIM technique allows for a direct assessment of the absorption and transmission behaviors, enabling a comprehensive evaluation of the metamaterial's performance in energy absorption and propagation. Numerical simulations were conducted to validate the working principle of the periodic structures, and the results were compared with experimental data to assess the accuracy of the TEOIM imaging system. By comparing TEOIM with other established methods, this study seeks to highlight the advantages and insights provided by TEOIM for metamaterial research.

## Materials and methods

### Measurement setup

**Figure 1:**
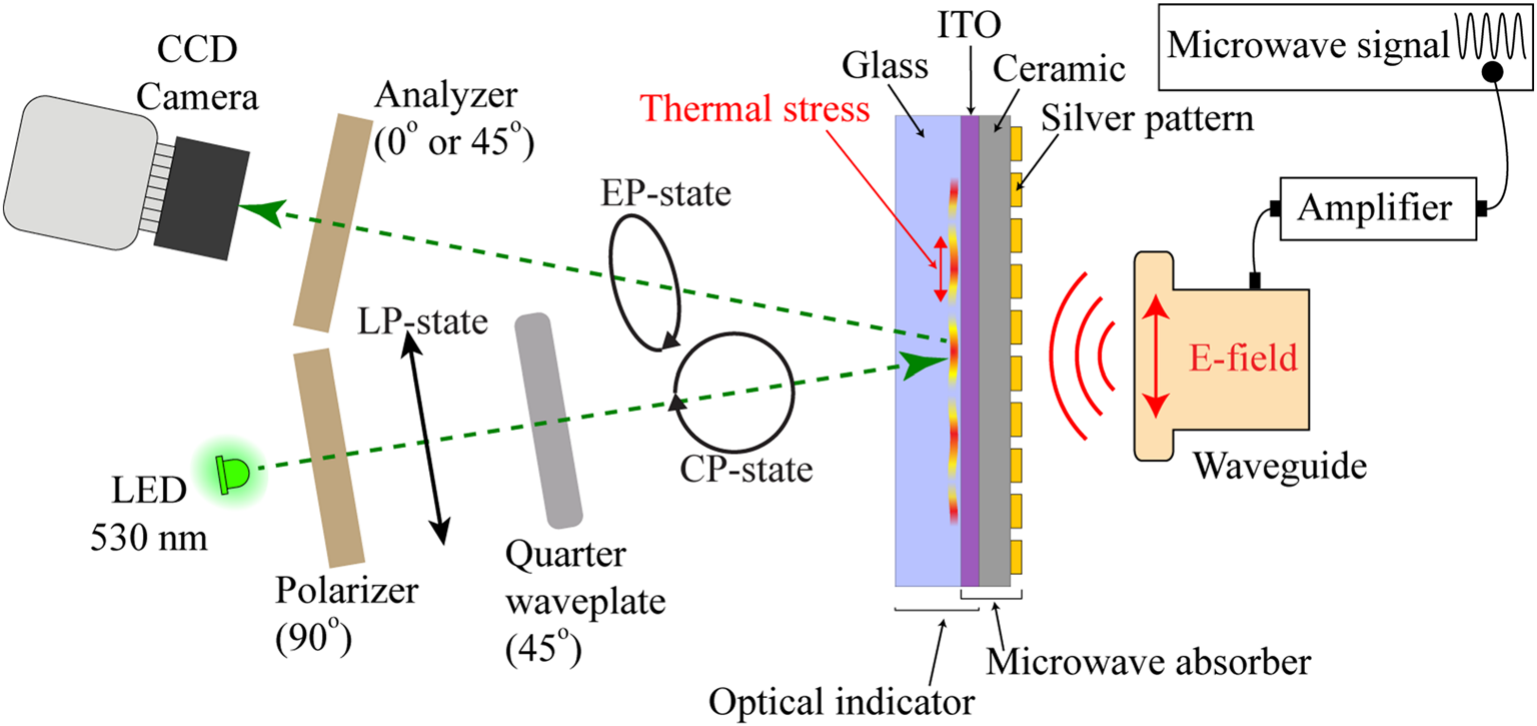
Illustration of the MWNF distribution measurement principle on the metasurface by the TEOIM setup. Probing green light is modulated to be circularly polarized using a linear polarizer and quarter waveplate. When the circularly polarized green light passes through the thermally stressed optical indicator, its polarization state becomes elliptically polarized due to the photoelastic effect emerging inside the glass substrate. After specular reflection, the CCD camera registers the light intensity via the analyzer (linear polarizer sheet) and visualizes the heat distribution corresponding to the initial H-MWNF distribution.

The TEOIM is characterized as a hybrid technology capable of visualizing the distribution of microwave electric and magnetic near-fields using the polarized light microscopy method^32,33^. Figure 1 shows the experimental setup and sample measurement configuration. The generated microwave signal (HP, 83620A) at 1 mW power is amplified up to 3 W using a power amplifier (Mini-Circuits, ZVE-3W-183+) and transmitted by a rectangular waveguide (Pasternack, WR-90, TE mode). The recommended frequency range for the WR-90 (10.16 mm × 22.86 mm aperture) waveguide covers 8.2–12.4 GHz. Our experiments are mainly focused on this range, which matches the X-band (8–12 GHz), where radar and satellite communication and wireless computer network technology operate. The light was emitted from a LED ($\lambda =530\mathrm{ nm}$) and was polarized circularly, passing through a linear polarizer (0°) and liquid crystal modulator (45°). ITO glass acts as an optical indicator (OI) for the visualization system, composed of the borosilicate Eagle XG (0.7 mm) glass substrate coated by an ITO (100 nm) thin layer. Since ITO is an electrically high conductive material, it will strongly interact with microwaves under electromagnetic radiation^34,35^. Because of the interaction and the microwave heating process, the ITO layer heats up, and the generated heat disseminates to the glass substrate causing local thermal stresses inside the glass. Moreover, the glass is a thermo-elastic medium, and due to mechanical stresses, the circularly polarized (CP) light passing through that material becomes elliptically polarized (EP) after specular reflection from the ITO layer. This phenomenon is known as photoelasticity, when a material's optical properties change under mechanical deformation.

In the final step, the CCD camera registers the light intensity through the analyzer, which is a second linear polarizer sheet oriented at 0 and 45 degrees successively, and the two images were stored for further image processing. The TEOIM microscope has a straightforward configuration setup and is not significantly sensitive to temperature fluctuations in the environment. The system is more sensitive under ambient light fluctuations. The measurement was implemented in a dark environment to prevent these alternative light noises, but the system is not thermally isolated. The microwave heating emerging in the glass substrates is very local and related to field distribution. Room temperature variation has no significant effect on the measurement processes, which was ignored during the experiments.

### Imaging process

The detected intensity of the two images describes the normal and shear stress distribution of the OIs, and they represent the linear birefringent (LB) *β*_1_ and *β*_2_ of the thermo-elastic medium. Therefore, the initial temperature and stress distribution of the OI causing those thermal deformations can be calculated by the following equation^32,36^:

1 $$\frac{q(x,y)}{C}= \left(\frac{{\partial }^{2}{\beta }_{1}(x,y)}{\partial {x}^{2}}-\frac{{\partial }^{2}{\beta }_{1}(x,y)}{\partial {y}^{2}}\right)+2\left(\frac{{\partial }^{2}{\beta }_{2}(x,y)}{\partial x\partial y}\right),$$

where *q* is the heat distribution, and *C* is the constant parameter correlated to the OI's material properties and the wavelength of the incident light. Initially, the CCD camera captures the first 100 (20 FPS) frames without applying a microwave signal, and all those images are accumulated and averaged as a background image. The subsequent 100 frames were captured when the microwave signal was applied to the metamaterial, and all that images were averaged as a data image. In the final step, the background image was subtracted from the data image to remove all instrumental and environmental noises. After each measurement cycle, about 5 s were delayed for the OI's cooling and thermal equilibrium, and these procedures were repeated 10 times to get a clearer image during visualization. Therefore, each experimental result represents the average of the image stack. A custom-made LabView program was used to control all devices and processes. Additionally, the images were smoothed with an algorithm, where a moving average of 25 pixels is tracked along the entire images (1024 × 768) to reduce a noise level caused by a local intensity fluctuation and possible environmental instability. More detailed information about the working principles of the TEOIM and image processing is included in Ref^32^.

### Optical indicators

The critical component of the TEOIM is an OI, and depending on the property of the indicator, it is possible to visualize either the electric or magnetic field distribution. Microwave heating, in the case of non-magnetic materials, has two primary loss mechanisms known as dielectric and resistive losses^37^. Dielectric loss emerges due to the electrical energy dissipation of insulating material by the time-varying electric field. Resistive losses are significant in high-conductive materials such as metals, metallic-based materials, and semiconductors. The time-varying magnetic field applied to a conductive material induces current flow, and the conductor starts to heat due to the Joule heating process^38^. Suppose the heating mechanism of the OI is based on the dielectric loss. In that case, it will heat up by a microwave electric field. Otherwise, if the dominant loss mechanism of the OI is resistive loss, it will visualize the H-MWNF distribution^36^. In the case of uniform ITO-coated glass, a conductive layer strongly interacts with a microwave magnetic field^39^, allowing visualization of the magnetic H-MWNF distribution. Besides the ITO glass, other highly conductive materials, such as gold, silver, platinum, and aluminum, can be applied as coatings on the thermo-elastic medium to serve as OI for the visualization of H-MWNF distribution^32^. In these specific measurements, standard ITO glass was utilized for the visualization, but it is important to note that the choice of optical indicators is not restricted to ITO glass alone. Note that TEOIM can also visualize electric field components using other OIs based on thin-layered materials with high dielectric losses^36^.

### Preparation of samples

For the fabrication of optically opaque metamaterial, a ceramic plate with 60 mm × 60 mm × 0.38 mm dimensions was used as a dielectric substrate. One side of the ceramic substrate was covered by a thin layer (about 35 μm) of silver sintering paste, and the sample was dried at 100 °C for 20 min. After that, the periodic structure was patterned by using a laser ablation technique. Finally, the patterned substrate was sintered for 120 min in two steps to remove the resin of the silver paste. In the first step, the sample was under 600 °C thermal condition for 60 min and the second at 880 °C for 60 min. Likewise, for the fabrication of transparent FSS, commercially available and uniform ITO glass (60 mm × 60 mm × 0.7 mm) was patterned by the same laser ablation procedure.

### Simulation

**Figure 2:**
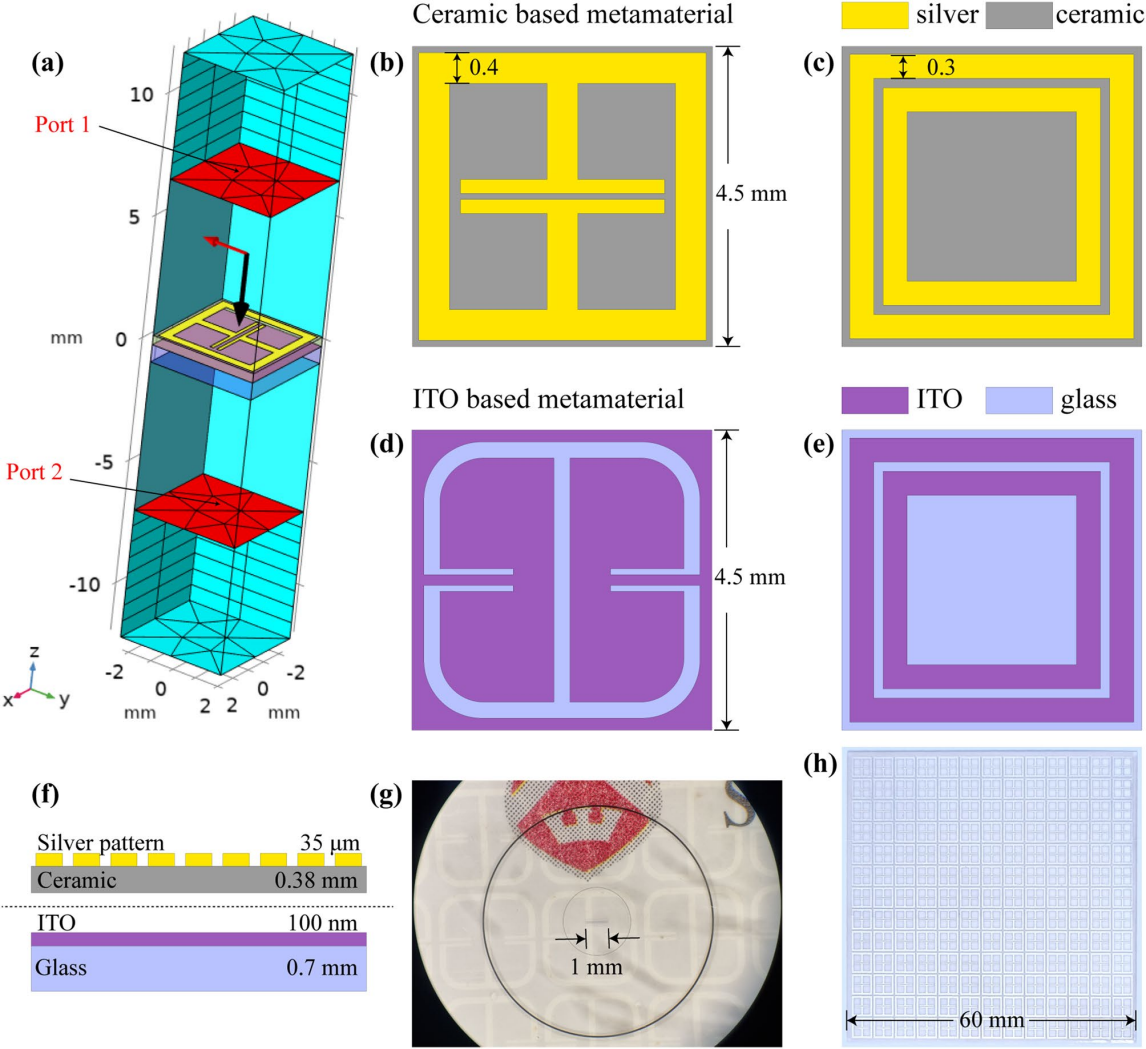
**(a)** Simulation model where the red-colored planes represent the ports. The red vector shows the polarization direction of the incident electromagnetic field, and the black is a Poynting vector. The geometry of the metasurface structure for **(b)** Ab-1, **(c)** Ab-2, **(d)** FSS-1, and **(e)** FSS-2. **(f)** Illustration of the side view of the uniform ITO glass and ceramic-based metamaterial layers. **(g)** The optical image of the ITO-based transparent FSS-1 sample under a microscope with a stage micrometer. **(h)** The photograph of the ceramic-based Ab-1 metamaterial.

Since the TEOIM is a visualization system based on the electromagnetic interaction and heating phenomenon of the thin metal, it involves complex physical processes and many nuances for a comprehensive understanding of all the mechanisms. It is even more challenging when the microwave and sample interaction happens with materials having complex geometrical shapes. Instead of classical theoretical analysis, modern computer simulation technologies are excellent solutions for such complex problems, and they are alternative ways for a detailed understanding of a specific design and present an optimization opportunity. Before the fabrication of the samples, the finite element method (FEM)-based COMSOL Multiphysics software was used for the design and detailed numerical analysis of the metamaterial. The simulation model consists of a multiphysics solution involving two physics interfaces: “*Electromagnetic Waves, Frequency Domain*” and “*Heat Transfer in Solids*.” The unit cell of the user-specified periodic structure is shown in Fig. 2a, where floquet-periodic boundary conditions are applied on the model walls. The *“Transition Boundary Condition”* was used to define interior boundaries to model a sheet of ITO and silver conductive layers. Numerical analysis was conducted to examine the structure’s electric and magnetic field distributions under normal incident *x*- and *y*-polarized plane waves and also to analyze the electromagnetic properties of the design. Two ceramic-based absorbers were designed for further investigation and labeled as Ab-1 (Fig. 2b) and Ab-2 (Fig. 2c), where 1 and 2 are for different samples, and “Ab” stands for the term “absorber”. Additionally, two ITO-based FSS are designed and labeled as FSS-1 (Fig. 2d) and FSS-2 (Fig. 2e). Ab-1 and Ab-2 are symmetric structures with a ceramic substrate patterned by silver elements with sub-wavelength size.

In general, metamaterial absorbers consist of two layers of periodic metal structures, termed front and back, separated by a dielectric substrate. However, in the case of an experiment by a TEOIM, the absorbers combine two physical components: a patterned ceramic substrate and a uniform ITO layer (Fig. 2f). These two components were attached face-to-face without an air gap during the visualization, as illustrated in Fig. 1. The metallic and uniform ITO thin layer acts as an OI for the visualization system. Simultaneously it becomes a background metallic layer for a ceramic-based absorber. Briefly, the absorber comprises the uniform ITO layer, ceramic substrate (*ε*_*r*_ = 9.8), and a patterned silver structure. The analysis includes the electric/magnetic field norm on the metasurface depending on the polarization of incident plane waves and absorption, reflection, and transmission spectra of the designed metamaterial related to the angular frequency. It can be calculated by the following equation:

2 $$A\left(\omega \right)=1-R\left(\omega \right)-T\left(\omega \right),$$

where $A\left(\omega \right)$, $R\left(\omega \right)={\left|{S}_{11}\right|}^{2}$, and $T\left(\omega \right)={\left|{S}_{21}\right|}^{2}$ are the absorption, reflection, and transmission magnitude corresponding to a certain frequency range. In the case of FSS, it does not contain ceramic-based periodic layers. Instead, they are single-layer metasurfaces directly patterned on the uniform ITO-coated glass. At the same time, patterned ITO-coated glass functions as a complete FSS and OI for a TEOIM. There is no need for an extra ceramic layer in the case of FSS. In the simulation model, characteristic parameters for modeling the ITO layer were defined with thickness* d* = 100 nm, density* ρ* = 7120 kg/m^3^, thermal conductivity* K* = 4 W/(m·K), specific heat capacity* C*_*p*_ = 341 J/(kg·K) and electrical conductivity* σ* = 1.25 × 10^6^ (S/m)^40^. Corning Eagle XG (solid) material was used from COMSOL Material Library to model the OI's glass substrate, and the relative permittivity was defined as *ε*_*r*_ = 5.27. All models were built corresponding to the actual experimental conditions. Figure 2g, h show the FSS-1 ITO-based transparent metasurfaces under a microscope and a photograph of the Ab-1 ceramic-based absorber, respectively.

## Results and discussion

**Figure 3:**
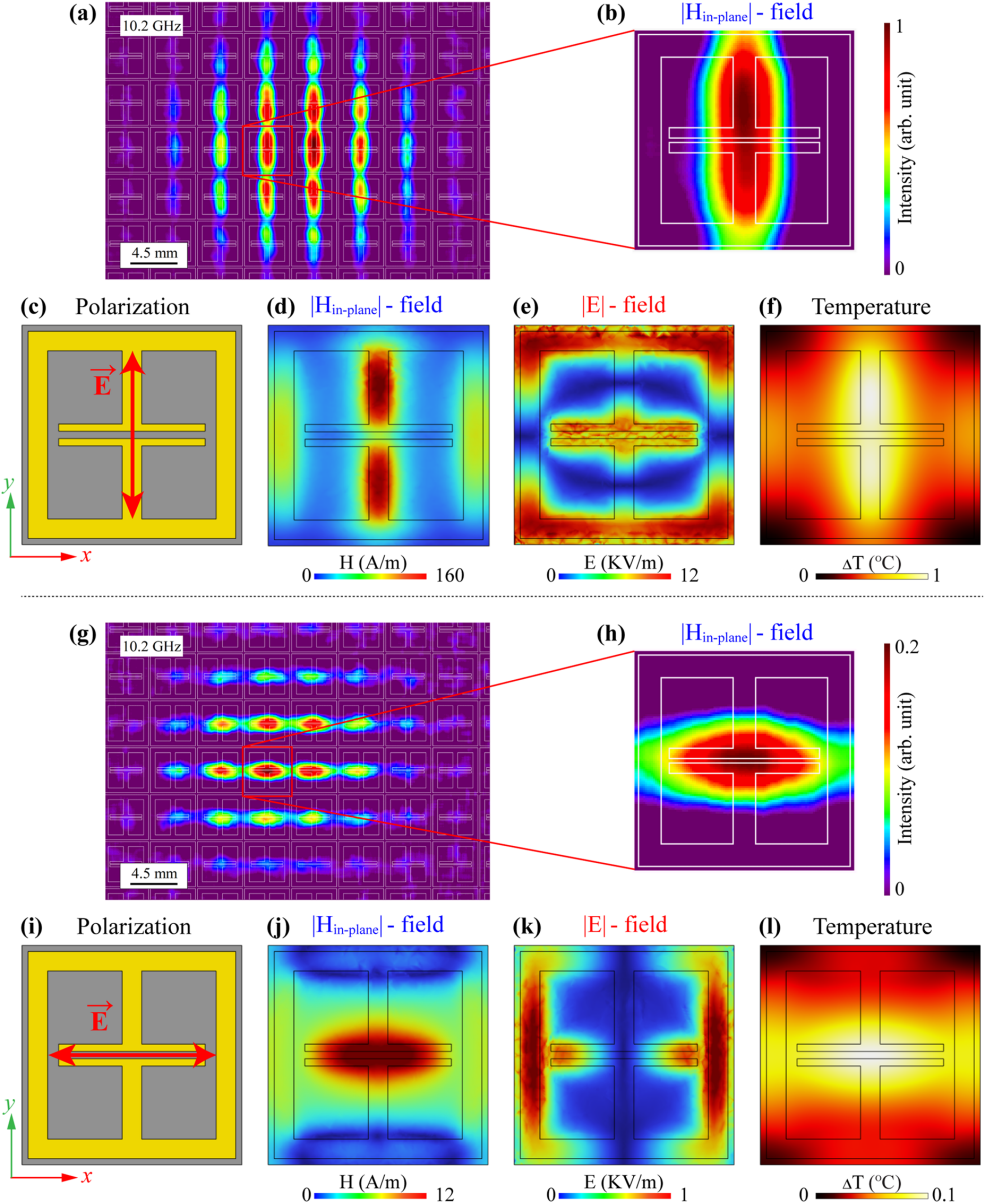
**(a)** Results of visualized H-MWNF distributions on the Ab-1 metasurface under the *E*_y_ polarization of the incident microwave field at 10.2 GHz **(a)** in a wide field of view and **(b)** in the unit cell. **(c)**, **(i)** The geometry of the unit cell with the polarization direction of the incident electromagnetic field. The simulation result on the unit cell at 10.2 GHz for (**d**) in-plane magnetic field distribution (|*H*_in-plane_|), (**e**) electric field distribution (|*E*|), and (**f**) thermal distribution under the *E*_y_ polarization of incident microwave. Results of visualized H-MWNF distributions on the same metasurface under the *E*_x_ polarization of the incident microwave field at 10.2 GHz **(g)** in a wide field of view and **(h)** in the unit cell. The simulation result on the unit cell at 10.2 GHz for (**j**) in-plane magnetic field distribution (|*H*_in-plane_|), (**k**) electric field distribution (|*E*|), and (**l**) thermal distribution under the *E*_x_ polarization of incident microwave.

The designed and optimized samples were fabricated for characterization by a TEOIM imaging technique. Figure 3 represents the experimental and simulation results for an Ab-1 absorber. All samples have a 4.5 mm unit cell size, and there are 169 unit cells on the ceramic surface. One of the advantages of the TEOIM is an extensive field of view that allows one to directly visualize the H-MWNF distribution for a relatively significant number of unit cells. Figure 3a shows the in-plane H-MWNF distribution on the Ab-1 absorber under the *y*-polarized incident plane wave at 10.2 GHz. The WR-90 waveguide is located directly on the center of the metamaterial with about a 10 mm distance between the metamaterial and the front of the waveguide, which is the reason that the field is distributed relatively more intense on the central cells. Figure 3b indicates the zoomed-in field distribution of one unit cell, and white lines highlight the structure's geometry.

Figure 3c shows the polarization direction of the incident plane waves relative to a designed simulation model. Figure 3d–f are simulation results corresponding to the in-plane H-MWNF distribution, electric field distribution, and thermal distribution under the *y*-polarization of the incident microwave at the same 10.2 GHz resonant frequency, respectively. The ITO plane was chosen to illustrate the simulated field distribution since the TEOIM system operates due to the ITO layer of the optical indicator. According to the results, the incident microwave intensely interacts with the designed geometry, and it is evident that visualized field distribution correctly matches an in-plane magnetic field distribution. Furthermore, the simulation indicates that the location where the structure experiences a temperature increase (ΔT = 1 °C) is comparable to the magnetic field distribution.

The waveguide was then rotated by 90°, and the MWNF distribution was visualized under the *x*-polarized plane wave, and the result is shown in Fig. 3g. Figure 3h depicts the zoomed-in single unit cell H-MWNF distribution. The polarization direction of the incident microwave relative to a metamaterial geometry is shown in Fig. 3i. Figure 3j–l are simulation results of field intensities under *x*-polarized plane waves at 10.2 GHz corresponding to the in-plane H-MWNF distribution, electric field distribution, and thermal distribution, respectively. Based on the results, the simulation shows that electric and magnetic fields have entirely different patterns, and it is apparent that the experimental result perfectly matches the in-plane H-MWNF distribution again. Compared to the *y*-polarized plane waves, the structure is weakly heated under the *x*-polarized incident plane wave (ΔT = 0.1 °C). Still, in this case, the thermal distribution of the barely heated metamaterial corresponds to a magnetic field. The TEOIM thus allows the investigation of the MWNF distribution on the metasurface depending on the polarization direction of incident waves and can improve the real-condition metamaterials characterization.

**Figure 4:**
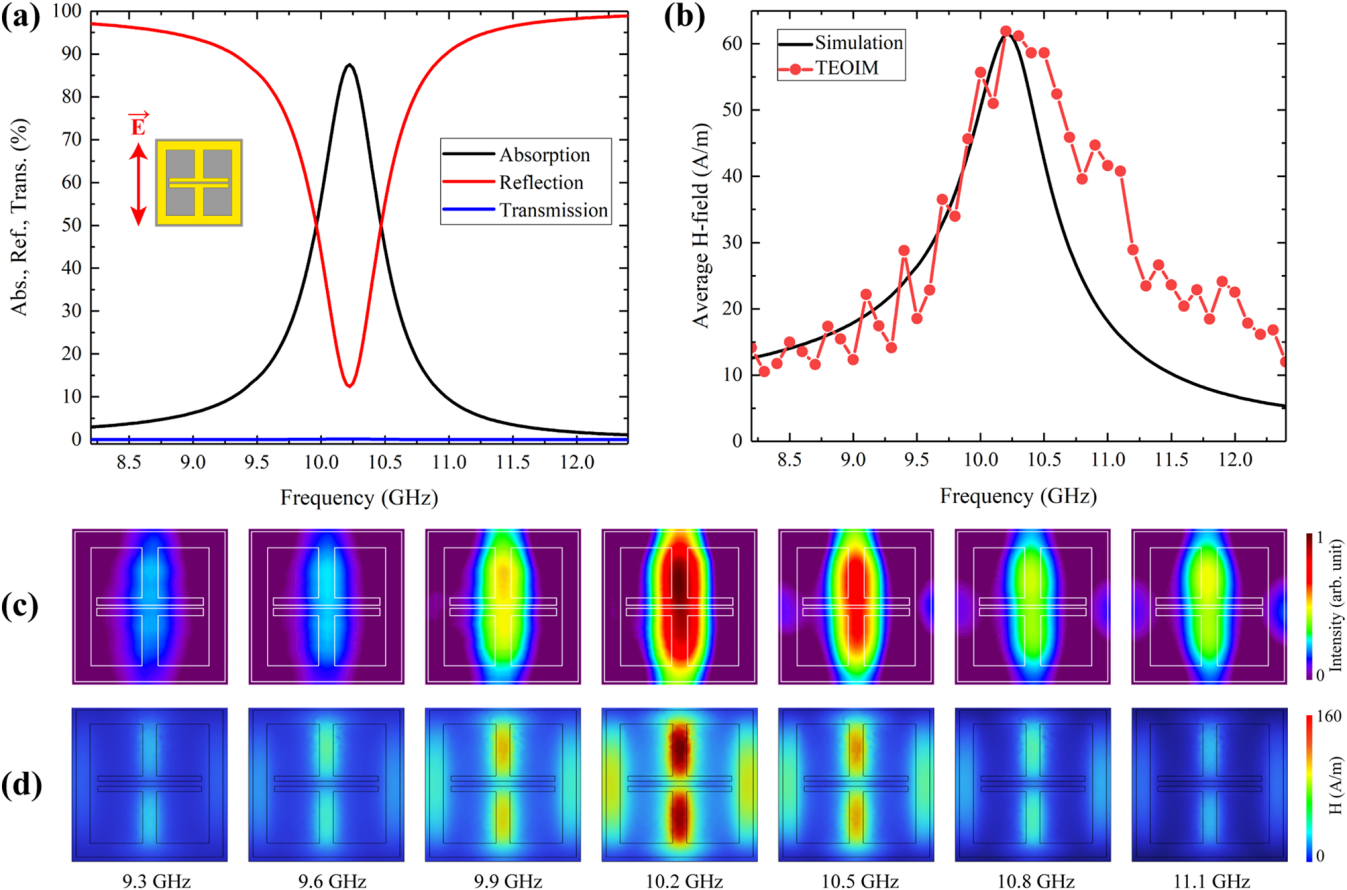
**(a)** Simulation results of the absorption, reflection, and transmission spectra of the Ab-1 absorber under *E*_y_-polarized plane waves. **(b)** Simulation results of average magnetic field intensity compared with an average intensity of visualized H-MWNF distribution. The results of H-MWNF distribution for different frequencies with a 0.3 GHz step around the resonance, obtained through **(c)** experiment and **(d)** simulation.

Figure 4a shows the simulation results of the designed metamaterial's absorption, reflection, and transmission spectra with about 85% absorption under *y*-polarized incident plane waves. Note that under the *x*-polarized plane waves, the Ab-1 sample does not absorb microwave energy, and the main focus was therefore placed only on this configuration. Numerical analysis shows that the absorption property of the metamaterial also can be evaluated by the magnetic or electric field intensity. The black line in Fig. 4b shows the simulation results of the average magnetic field intensity of the unit cell. Compared with the absorption curve, it is noteworthy that the shapes are slightly different, but the resonant frequencies match perfectly.

Consequently, the magnetic field has the highest intensity when the Ab-1 absorber has the maximum absorption. That can be essential in finding resonant frequencies or operating bandwidth through visualization techniques like a TEOIM. The Ab-1 absorber was investigated in the bandwidth of the waveguide by doing a frequency sweep with a 0.1 GHz step. As a result, all the field intensities were evaluated and averaged, which is shown in Fig. 4b (red line), and it adequately compares with the simulation result. It was tested to evaluate the intensity tendency by calculating the average field intensity of the visualized full images and the average field intensity of the chosen specific unit cell. The results revealed a very similar trend within the same frequency range. Therefore, the entire field of view from the images was selected for final averaging and evaluation of the field intensity, and the average field intensity data were rescaled using min–max normalization to establish a correlation with the simulation range. Figure 4c, d shows visualized and simulated H-MWNF distributions of the unit cell for several frequencies around the 10.2 GHz resonant frequency. The depicted distributions exhibit a remarkable correlation with the intensity trend and field shapes, indicating a good agreement between the visualized and simulated results. The image of 10.2 GHz shows maximum intensity where the Ab-1 sample has about 85% absorption at this frequency. The main difference between the curves is bandwidth, where in the case of simulation, the metamaterial has a narrow absorption bandwidth, while the experimental evaluation shows a wider bandwidth. The main reason for this is that the physical phenomenon of measurement where the imaging of the field distribution is implemented is based on thermal stress analysis, which is a thermal and optical phenomenon. It is reasonable to assume that the absorption or transmission evaluation based on the thermal energy evaluation could not be as accurate as specialized RF instruments. The number one priority of the TEOIM system is to provide visual information about metamaterials' interaction with electromagnetic waves.

**Figure 5:**
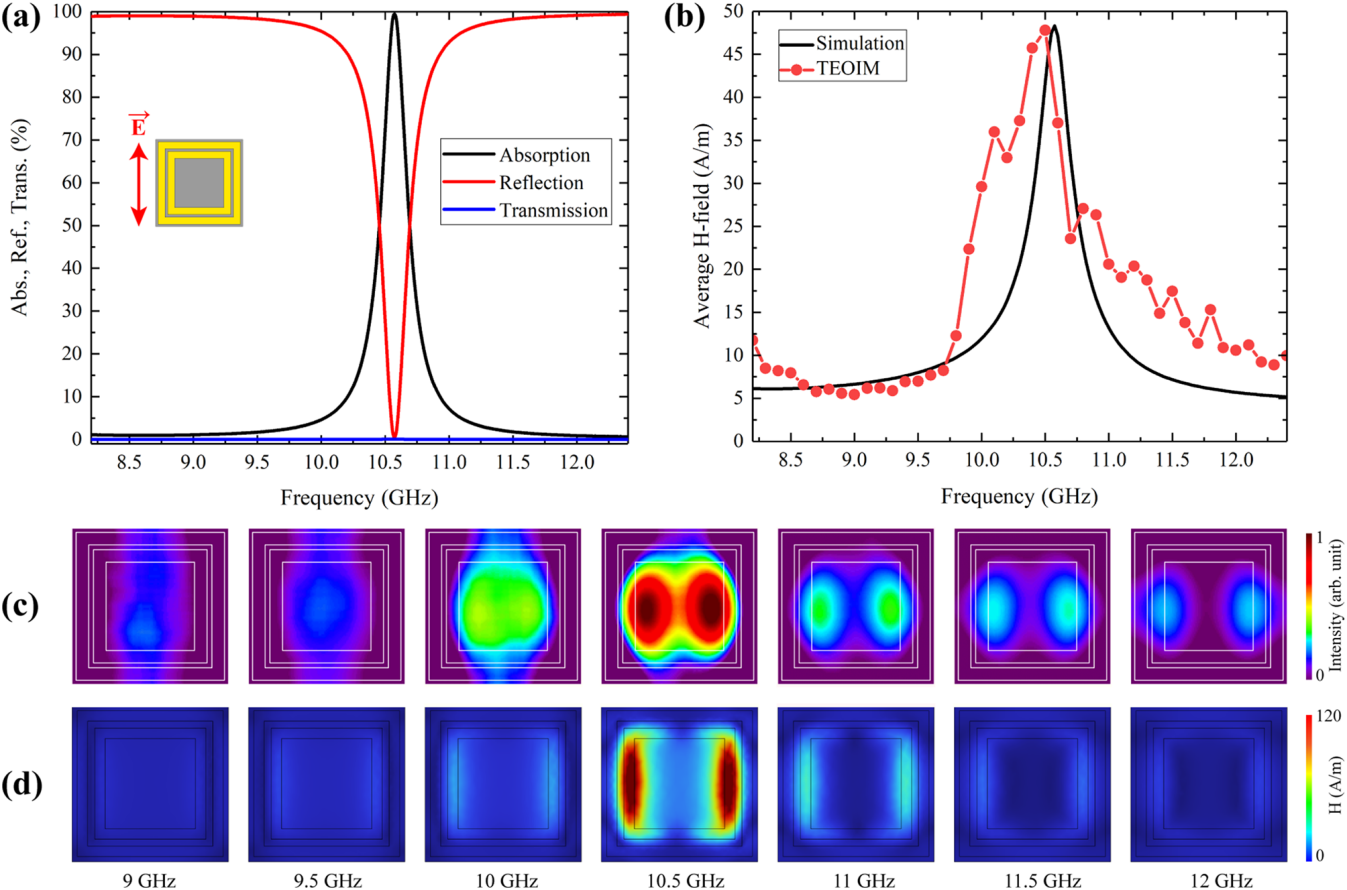
**(a)** Simulation results of the absorption, reflection, and transmission spectra of the Ab-2 absorber under *E*_y_-polarized plane waves. **(b)** Simulation results of average magnetic field intensity compared with an average intensity of visualized H-MWNF distribution. The results of H-MWNF distribution for different frequencies with a 0.5 GHz step around the resonance, obtained through **(c)** experiment and **(d)** simulation.

The second Ab-2 absorber comprises double square metallic loops with the same vertical and horizontal linear symmetry. This structure, with the composition of the same materials as Ab-1, has about 100% absorption for a 10.5 GHz frequency and a narrower bandwidth compared with the Ab-1 sample. Simulation results of the absorption reflection and transmission spectra for the Ab-2 sample are presented in Fig. 5a. The experimental results of the TEOIM were averaged considering the whole area of the camera's field of view, and the H-MWNF distribution intensity of the images was compared to the simulation result and is in good agreement, as presented in Fig. 5b. The experimental results are shifted with the simulation results about 0.1 GHz in the resonance frequency, and the bandwidth is slightly wider. Future improvement of the TEOIM sensitivity, with fewer fluctuations, higher stability, and smaller step size of frequency sweep, will give a more accurate characterization of the metamaterials. The visualized and simulated distributions of H-MWNF for various frequencies around the 10.5 GHz resonant frequency are depicted in Fig. 5c, d.

**Figure 6:**
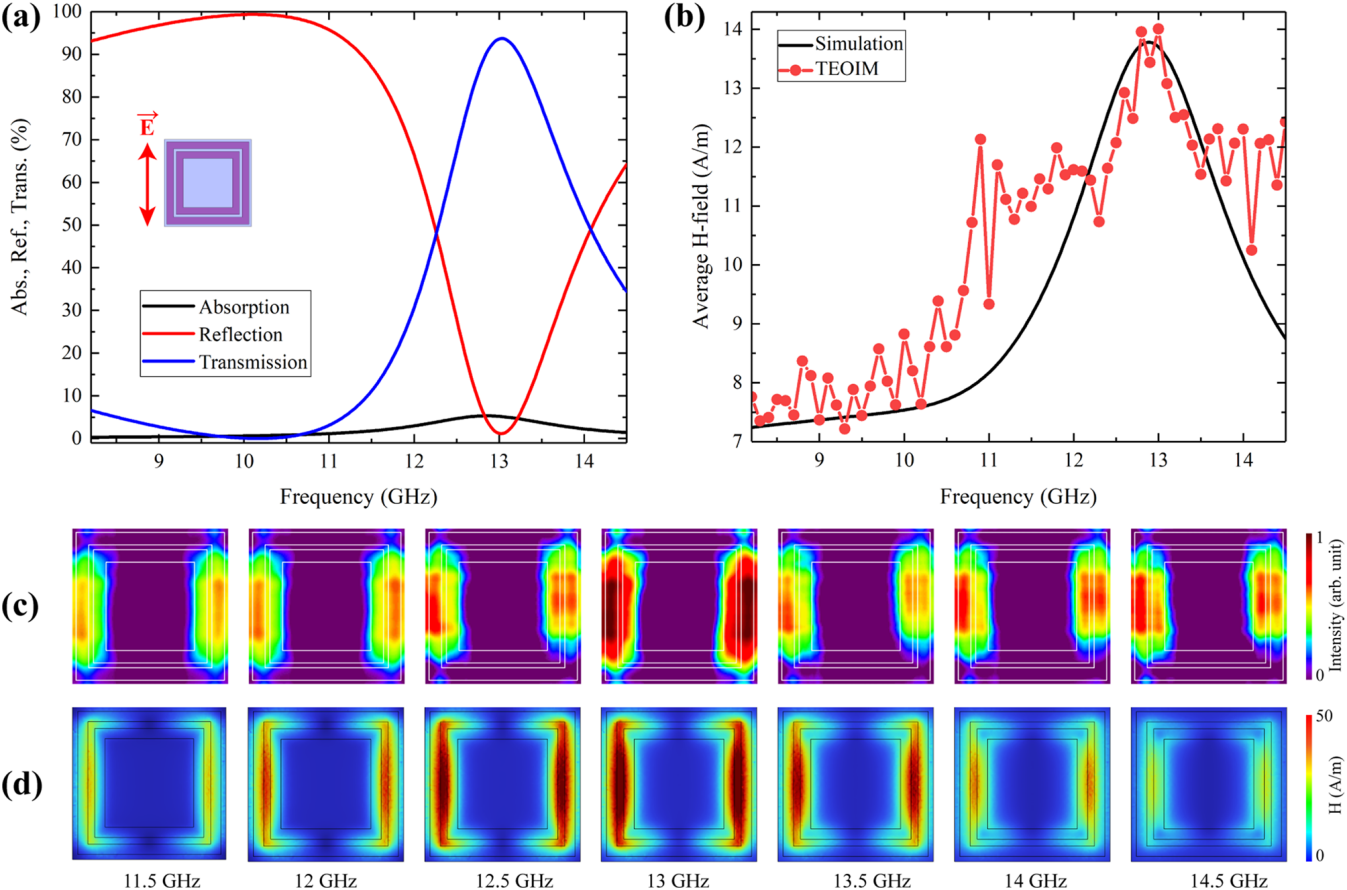
**(a)** Simulation results of the absorption, reflection, and transmission spectra of the FSS-2 metasurface under *E*_y_-polarized plane waves. **(b)** Simulation results of average magnetic field intensity compared with an average intensity of visualized H-MWNF distribution. The results of H-MWNF distribution for different frequencies with a 0.5 GHz step around the resonance, obtained through **(c)** experiment and **(d)** simulation.

FSS structures generally operate based on the bandpass or bandstop response and filter or block electromagnetic waves of a required frequency^41,42^. Besides the absorbers, two single-layer FSS were designed and tested by a TEOIM. The double square metallic loops with the same geometry were directly patterned on the uniform ITO glass, and it became a bandpass filter for around 13 GHz frequencies. An additional rectangular waveguide Pasternack WR-62 (12.4–18 GHz) was used in the experiment to extend the operating frequency band. Simulation results of FSS-2 are presented in Fig. 6a, and it shows that about 95% of the incident microwave can transmit through the metasurface around 13 GHz frequency with 1.2 GHz bandwidth; otherwise, it is a reflective layer. According to the simulation results, the magnetic field intensity on the unit cell can characterize the transmission property of the FSS metasurfaces, similar to the absorption characterization in the case of metamaterial absorbers. The black line in Fig. 6b indicates the simulation results of the average magnetic field intensity for unit cells, where the shape of the curve is again slightly different, but the peak frequency again matches the peak of the transmission curve. The visualized and simulated H-MWNF distribution of the unit cell is shown in Fig. 6c, d around the central frequency of 13 GHz with a 0.5 GHz step for the FSS-2 sample. Although the FSS-2 and Ab-2 samples have the same geometry, the field distribution is somewhat different. The reason for this is the ceramic substrate. In the case of the FSS-2 sample, the H-MWNF distribution images directly show the field on the plane of the periodic structure. However, in the case of the Ab-2 absorber between the periodic silver structure and the uniform ITO glass (OI), there is a ceramic plate with a 0.38 mm thickness. Therefore, during the transmission through the dielectric substrate, the shape of the H-MWNF distribution partially blurs, tending to move the central part of the cells. Alternatively, in the case of the Ab-2 absorber, it can be due to the strong coupling between the absorber's metallic silver pattern and the background ITO layer. The experimental curve in Fig. 6b shows a higher fluctuation: this may be due to the roughly split surface of the OI, which is also a metasurface. Since the TEOIM uses the reflected light from the OI to visualize the H-MWNF distribution, it requires a higher reflective surface fraction for proper visualization. Therefore, the FSS-1 metasurface was designed to have a more metallic surface fraction to ensure a high enough reflected light intensity from the metasurface. The numerical analysis includes the absorption, reflection, and transmission spectra, the electric and magnetic field norm on the unit cell, and the thermal distribution.

**Figure 7:**
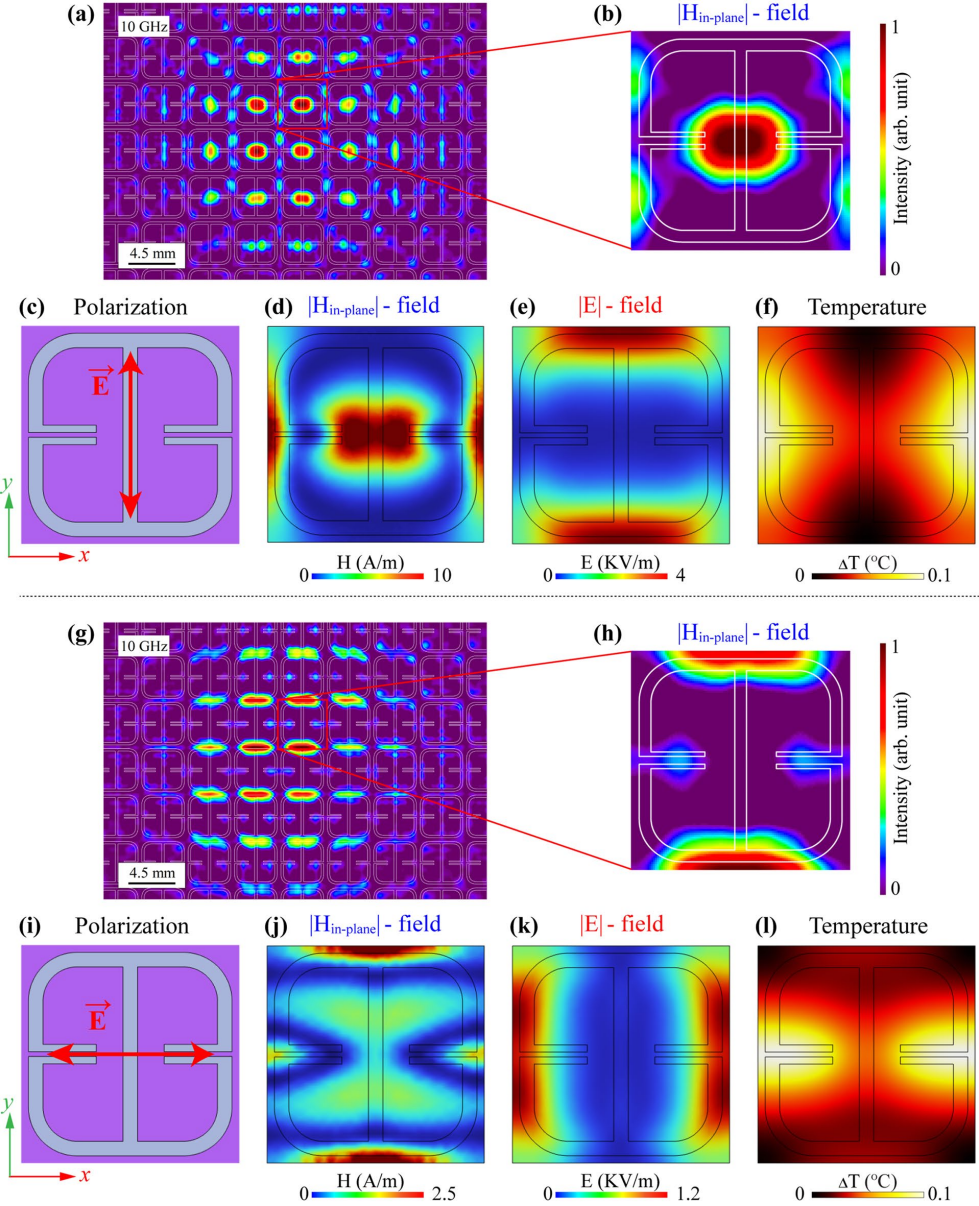
**(a)** Results of visualized H-MWNF distributions on the FSS-1 metasurface under the *E*_y_ polarization of the incident microwave field at 10 GHz **(a)** in a wide field of view and **(b)** in the unit cell. **(c)**, **(i)** The geometry of the unit cell with the polarization direction of the incident electromagnetic field. The simulation result on the unit cell at 10 GHz for (**d**) in-plane magnetic field distribution (|*H*_in-plane_|), (**e**) electric field distribution (|*E*|), and (**f**) thermal distribution under the *E*_y_ polarization of incident microwave. Results of visualized H-MWNF distributions on the same metasurface under the *E*_x_ polarization of the incident microwave field at 10 GHz **(g)** in a wide field of view and **(h)** in the unit cell. The simulation result on the unit cell at 10 GHz for (**j**) in-plane magnetic field distribution (|*H*_in-plane_|), (**k**) electric field distribution (|*E*|), and (**l**) thermal distribution under the *E*_x_ polarization of incident microwave.

Figure 7 illustrates all experimental and simulated field distributions depending on the polarization of incident microwaves. Figure 7a shows H-MWNF distribution on FSS-1 with a wide field of view under *E*_y_ polarized incident microwave, and Fig. 7b depicts the field on only one unit cell. In the central location of the cell, the field is localized more intensively. Figure 7c is a geometry of the unit cell from the simulation model relative to the E-vector, and Fig. 7d–f represent the in-plane H-MWNF distribution, electric field distribution, and thermal distribution, respectively. The second section of Fig. 7 describes the case of the *x*-polarized incident microwave. The experimental result is shown in Fig. 7g and its zoomed H-MWNF distribution on the unit cell (Fig. 7h).

**Figure 8:**
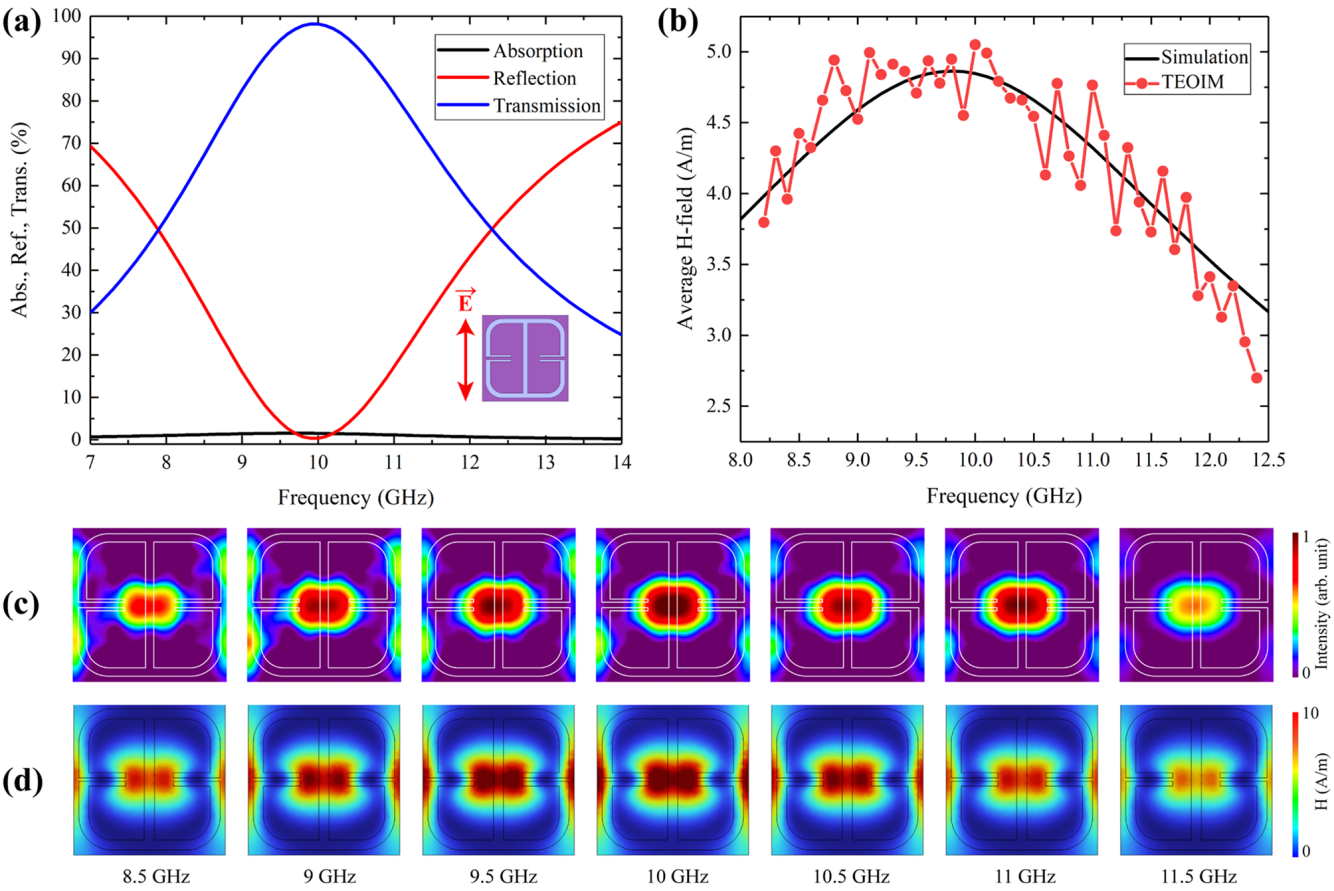
**(a)** Simulation results of the absorption, reflection, and transmission spectra of the FSS-1 metasurface under *E*_y_-polarized plane waves. **(b)** Simulation results of average magnetic field intensity compared with an average intensity of visualized H-MWNF distribution. The results of H-MWNF distribution for different frequencies with a 0.5 GHz step around the resonance, obtained through **(c)** experiment and **(d)** simulation.

The last line of the images is a simulation result where Fig. 7i illustrates the polarization direction of the E-vector, and Fig. 7j–l describe the in-plane H-MWNF distribution, electric field distribution, and thermal distribution, respectively. Similar to the Ab-1 absorber, in the case of FSS-1, it is clear that visualized result by a TEOIM corresponds to the H-MWNF distribution. FSS-1 sample functions effectively as a bandpass filter, and as expected in both polarization cases, thermal distribution results show negligible temperature differences (ΔT = 0.1 °C; Fig. 7f, l). In contrast, metamaterial absorbers such as the Ab-1 sample, the absorbed microwave energy predominantly converts into thermal energy (ΔT = 1 °C), as depicted in Fig. 3f. Analysis of microwave characteristics of the single-layer FSS-1 metamaterial is presented in Fig. 8a. The designed metamaterial has near zero absorption and operates as a bandpass filter around the 10 GHz central frequency region with 3 GHz bandwidth. The experimental and simulation results of the average intensity of the H-MWNF distributions are presented in Fig. 8b. Based on the outcome, for FSS metamaterials, the field intensity is higher when the transmission of the incident microwave power is highest. Visualized and simulated images of the H-MWNF distribution for several frequencies with a 0.5 GHz step around the 10 GHz central frequency are presented in Fig. 8c, d.

One of the best experimental methods for metamaterial characterization is an over-the-air (OTA) measurement equipped with a vector network analyzer (VNA) by simply placing the sample in the middle between two antennas and performing a sweep over the required frequency band^43–45^. Compared to this traditional method, a TEOIM measurement of absorption, transmission and reflection is somewhat slower. However, with the TEOIM, visual information about field information is obtained, which allows the investigation of a structure’s operating principles in real conditions. Furthermore, the system can easily distinguish the damaged cells via the MWNF pattern if the fabricated materials have some defects or deviations.

The presented results do not limit applications of the TEOIM, and more accurate characterization can be achieved by increasing the camera's sensitivity and speed, the field of view of the system, and by using advanced optics and sensors. In addition, this equipment is expected to be a valuable testing solution for composite material and metamaterial characterization in the near future.

While investigating H-MWNF distribution on 2D metamaterials may not directly represent the complexity of 3D metamaterials, it serves as a valuable starting point for understanding and designing advanced materials. The insights from studying 2D systems can be applied and extrapolated to develop more intricate 3D metamaterials with tailored electromagnetic properties for various practical applications. The TEOIM method can also be used to gain insights into the field structure in the near region of the complex 3D metamaterial surface. It is fundamental for understanding 3D metamaterial behavior under microwave interaction. Moreover, the TEOIM system can visualize and reconstruct the 3D bulk images around any metamaterial structure by sequentially changing the distance between the OI and metamaterial under test^36^. This process allows for systematically exploring different configurations and can lead to novel 3D metamaterial designs with desired electromagnetic properties. Since the TEOIM visualizes the field distribution due to the OI, which is a physical component, it can not investigate the microwave near field distribution inside the metamaterial layers. It is a practical high-resolution imaging method for characterizing the electromagnetic fields and properties on the outer layers of the metamaterials and near region around it.

Another rapidly evolving field is metamaterial coding, which holds great promise. Researchers are actively focused on exploring its potential and pushing the boundaries of this exciting area^46–49^. Metamaterial coding has diverse applications, from information encryption and authentication to data storage, wireless communication, radar technology, and optical and infrared cloaking^50,51^. Visualizing the electric or magnetic fields near the metamaterial surface will be crucial in characterizing metamaterial properties, designing optimized structures, and enabling information encoding and decoding by imaging methods. TEOIM can successfully enhance this research aspect. Additionally, the TEOIM provides notable advantages for characterizing non-symmetric metamaterials through electric or magnetic field visualization. Generally, these tasks are challenging for simulation methods because of the non-symmetric structure. For instance, certain metamaterials consist of cells that undergo slow rotation after each periodic step^52^, and numerical calculation for this kind of metamaterial having continuous rotational symmetry requires high computational power. Overall, the visualization of microwave metamaterials using TEOIM holds promise for advancing our understanding of their behavior and optimizing their performance.

## Conclusions

The TEOIM visualization system was applied to investigate the microwave interaction mechanism of metamaterials with electromagnetic waves. By designing various absorbers and FSS metamaterials, H-MWNF distribution was visualized using the TEOIM imaging technique. The evaluation of magnetic field intensity allowed the prediction of the metamaterials' absorption, transmission, and reflection properties. The experimental results were consistent with the corresponding simulation models, validating the effectiveness of the imaging system. The TEOIM system holds potential for further applications in the structural characterization and optimization of metamaterials. By leveraging its capabilities, researchers can gain deeper insights into the behavior and performance of metamaterials, paving the way for improved designs and enhanced functionalities. The TEOIM system offers exciting opportunities for advancing metamaterial research and development.

## Data Availability

The datasets generated and/or analyzed during the current study are available from the corresponding author on reasonable request.
